# Methods for analyzing cost effectiveness data from cluster randomized trials

**DOI:** 10.1186/1478-7547-5-12

**Published:** 2007-09-06

**Authors:** Max O Bachmann, Lara Fairall, Allan Clark, Miranda Mugford

**Affiliations:** 1 School of Medicine, Health Policy and Practice, University of East Anglia, Norwich, UK; 2 Lara Fairall, Research Fellow, University of Cape Town Lung Institute, University of Cape Town, Cape Town, South Africa

## Abstract

**Background:**

Measurement of individuals' costs and outcomes in randomized trials allows uncertainty about cost effectiveness to be quantified. Uncertainty is expressed as probabilities that an intervention is cost effective, and confidence intervals of incremental cost effectiveness ratios. Randomizing clusters instead of individuals tends to increase uncertainty but such data are often analysed incorrectly in published studies.

**Methods:**

We used data from a cluster randomized trial to demonstrate five appropriate analytic methods: 1) joint modeling of costs and effects with two-stage non-parametric bootstrap sampling of clusters then individuals, 2) joint modeling of costs and effects with Bayesian hierarchical models and 3) linear regression of net benefits at different willingness to pay levels using a) least squares regression with Huber-White robust adjustment of errors, b) a least squares hierarchical model and c) a Bayesian hierarchical model.

**Results:**

All five methods produced similar results, with greater uncertainty than if cluster randomization was not accounted for.

**Conclusion:**

Cost effectiveness analyses alongside cluster randomized trials need to account for study design. Several theoretically coherent methods can be implemented with common statistical software.

## Background

Cluster randomized trials are commonly used to evaluate the effectiveness and cost effectiveness of interventions in health care, health promotion and health professional education. Groups of individuals, such as doctors' patients or schools' pupils, are allocated together to receive different interventions or to follow usual practice. One key advantage of randomly allocating groups rather than individuals is that it permits inferences about the intervention's effects on service providers as well as on users. For example, in a trial of an educational intervention aimed at doctors, allocating doctors together with their patients permits inferences about the intervention's effects on doctors as well as on their patients. But cluster randomization tends to reduce the statistical power and precision of trials because of similarities between individuals within each cluster, compared to individuals in other clusters. This similarity, or non-independence, is expressed as an intra-cluster, or intra-class, correlation coefficient (ICC) [1]. The ICC is the proportion of response variance that occurs between clusters, as a proportion of the total variance (within and between clusters) [1]. Statistical methods for comparing outcomes in cluster randomized trials are now well developed [1,2]. But there has been little methodological research on appropriate methods for jointly analyzing cost and effectiveness data from cluster randomized trials [3,4].

**Figure 1:**
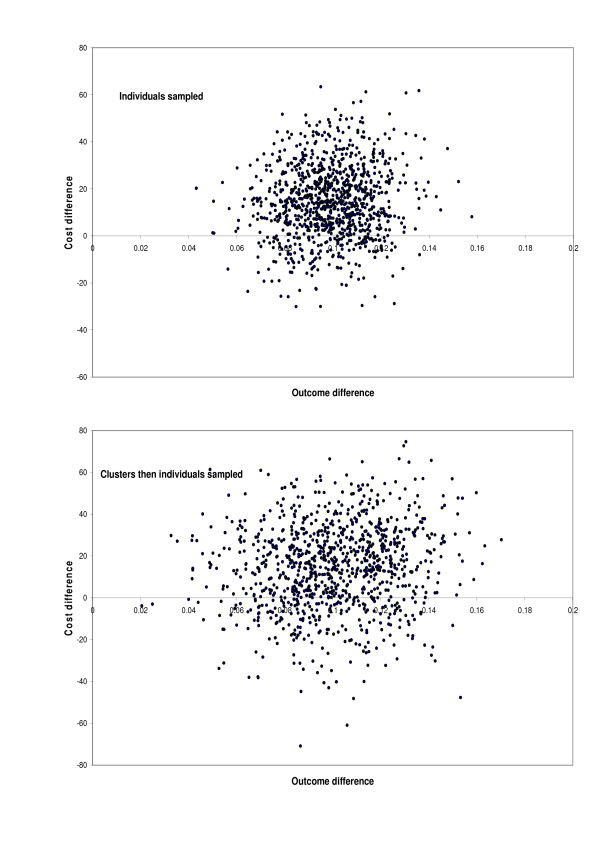
Cost effectiveness plane from bootstrap sampling of individuals, or clusters then individuals.

Cost effectiveness analysis with individual level costs and outcomes is more complex than analysis of effects or costs alone, because differences in costs and differences in outcomes need to be analysed together. Uncertainty about cost effectiveness estimates can be quantified as confidence intervals for incremental cost effectiveness ratios (ICERs), or probabilities that interventions are cost effective, using two general approaches [5]. One approach combines cost and effect data on a two dimensional cost effectiveness plane (for example, Figure 1) [5-7]. It models the cost difference in one dimension and the outcome difference in the other, taking into account their variances and covariance and producing a cost effectiveness ellipse. The other approach combines cost and outcome measures for each individual by calculating net benefits, which are then compared between trial arms [4,8,9]. Both general approaches can be used for cluster randomized trials [3,4].

Clustering of costs and of outcomes is often neglected in economic evaluations alongside cluster randomized trials that have individual cost data. Several economic evaluations alongside cluster randomized trials have used bootstrapping to deal with asymmetrically distributed costs [10-12], but without specifying whether they sampled individuals or clusters. If they simply sampled individuals then cluster randomization design effects would not have been accounted for. Other studies adjusted for clustering using hierarchical regression models to estimate cost differences and outcome differences and their variances [13,14]. They then used these values to plot confidence ellipses. But neither of these two studies estimated ICER confidence intervals or considered correlations between costs and effects. Others have adjusted for clustering when comparing costs and comparing outcomes, but without estimating ICERs or probabilities that the intervention was cost effective [15].

In this paper we describe several appropriate methods for analyzing cost effectiveness data from cluster randomized trials. We show how to apply these methods using data from one such trial [16].

## Example

This cluster randomized trial evaluated an educational intervention aimed at improving the management of lung disease in adults attending South African primary care clinics [16]. Forty clinics were randomized to intervention or control arms. In each clinic 50 patients were interviewed at baseline and 3 months later. The trial outcome, indicating appropriate care, was defined as present if a patient was 1) newly diagnosed as having tuberculosis or 2) treated with inhaled corticosteroids for asthma or 3) referred for higher level care in the presence of defined indicators of severe illness. Health service costs were measured for each subject and included costs of clinic and hospital attendance, investigations, drugs, ambulance transport and the educational intervention. Complete data were available on 1856 patients. The prevalence of desirable outcome was 21% in the intervention arm and 11% in the control arm (odds ratio 2.2, 95% CI adjusted for clustering 1.5–3.2, ICC = 0.061). Costs were positively skewed, with mean costs of 220 South African Rand (ZAR) (median 159, interquartile range 77–266) in the intervention arm and ZAR 205 (median 140, interquartile range 70–218) in the control arm, and ICCs of 0.01 in each arm. The point estimate for the incremental cost effectiveness ratio was thus ZAR150 per unit or effect ((ZAR 220–205)/(21%–11%)). Net benefits were not normally distributed. At willingness to pay of zero they were, by definition, the negative of costs, that is, with negative values and negatively skewed. At higher willingness to pay levels, net benefits were bimodal, depending on whether subjects had experienced the outcome or not. The ICC for net benefits ranged from 0.01 (at willingness to pay of zero) to 0.036 (at willingness to pay of ZAR2000). These ICCs indicated that clustering of costs and outcomes needed to be accounted for. The remainder of this paper shows how this can be done. For each method we outline the analytic principles, describe how they were implemented with Stata [17] or WinBUGS [18] software, and show the results.

## Methods

### 1. Joint modelling of costs and effects

#### 1.1 Bayesian parametric model

Nixon and Thompson have shown how to model costs and effects jointly for individually randomized trial data [7]. We have adapted the model to cluster randomized trials. We assume that we have two treatments which we wish to compare. In general, we assume that cost (*C*) and effectiveness (*E*) have distributions which can be characterised by their means and variances:

cijk~Dist(μijkC,σijkC)eijk~Dist(μijkE,σijkE)
 MathType@MTEF@5@5@+=feaafiart1ev1aaatCvAUfKttLearuWrP9MDH5MBPbIqV92AaeXatLxBI9gBaebbnrfifHhDYfgasaacH8akY=wiFfYdH8Gipec8Eeeu0xXdbba9frFj0=OqFfea0dXdd9vqai=hGuQ8kuc9pgc9s8qqaq=dirpe0xb9q8qiLsFr0=vr0=vr0dc8meaabaqaciaacaGaaeqabaqabeGadaaakeaafaqabeGabaaabaGaem4yam2aaSbaaSqaaiabdMgaPjabdQgaQjabdUgaRbqabaGccqGG+bGFcqqGebarcqqGPbqAcqqGZbWCcqqG0baDcqqGOaakiiGacqWF8oqBdaqhaaWcbaGaemyAaKMaemOAaOMaem4AaSgabaGaem4qameaaOGaeiilaWIae83Wdm3aa0baaSqaaiabdMgaPjabdQgaQjabdUgaRbqaaiabdoeadbaakiabcMcaPaqaaiabdwgaLnaaBaaaleaacqWGPbqAcqWGQbGAcqWGRbWAaeqaaOGaeiOFa4NaeeiraqKaeeyAaKMaee4CamNaeeiDaqNaeeikaGIae8hVd02aa0baaSqaaiabdMgaPjabdQgaQjabdUgaRbqaaiabdweafbaakiabcYcaSiab=n8aZnaaDaaaleaacqWGPbqAcqWGQbGAcqWGRbWAaeaacqWGfbqraaGccqGGPaqkaaaaaa@670D@

where μijkC
 MathType@MTEF@5@5@+=feaafiart1ev1aaatCvAUfKttLearuWrP9MDH5MBPbIqV92AaeXatLxBI9gBaebbnrfifHhDYfgasaacH8akY=wiFfYdH8Gipec8Eeeu0xXdbba9frFj0=OqFfea0dXdd9vqai=hGuQ8kuc9pgc9s8qqaq=dirpe0xb9q8qiLsFr0=vr0=vr0dc8meaabaqaciaacaGaaeqabaqabeGadaaakeaaiiGacqWF8oqBdaqhaaWcbaGaemyAaKMaemOAaOMaem4AaSgabaGaem4qameaaaaa@33BC@ is the mean cost for the *i*^th ^the individual in the *j*^th ^cluster in arm *k *of the trial and σijkC
 MathType@MTEF@5@5@+=feaafiart1ev1aaatCvAUfKttLearuWrP9MDH5MBPbIqV92AaeXatLxBI9gBaebbnrfifHhDYfgasaacH8akY=wiFfYdH8Gipec8Eeeu0xXdbba9frFj0=OqFfea0dXdd9vqai=hGuQ8kuc9pgc9s8qqaq=dirpe0xb9q8qiLsFr0=vr0=vr0dc8meaabaqaciaacaGaaeqabaqabeGadaaakeaaiiGacqWFdpWCdaqhaaWcbaGaemyAaKMaemOAaOMaem4AaSgabaGaem4qameaaaaa@33C9@ is the standard deviation of the cost. Similarly μijkE
 MathType@MTEF@5@5@+=feaafiart1ev1aaatCvAUfKttLearuWrP9MDH5MBPbIqV92AaeXatLxBI9gBaebbnrfifHhDYfgasaacH8akY=wiFfYdH8Gipec8Eeeu0xXdbba9frFj0=OqFfea0dXdd9vqai=hGuQ8kuc9pgc9s8qqaq=dirpe0xb9q8qiLsFr0=vr0=vr0dc8meaabaqaciaacaGaaeqabaqabeGadaaakeaaiiGacqWF8oqBdaqhaaWcbaGaemyAaKMaemOAaOMaem4AaSgabaGaemyraueaaaaa@33C0@ is the mean effectiveness for the *i*^th ^the individual in the *j*^th ^cluster in arm *k *of the trial and σijkE
 MathType@MTEF@5@5@+=feaafiart1ev1aaatCvAUfKttLearuWrP9MDH5MBPbIqV92AaeXatLxBI9gBaebbnrfifHhDYfgasaacH8akY=wiFfYdH8Gipec8Eeeu0xXdbba9frFj0=OqFfea0dXdd9vqai=hGuQ8kuc9pgc9s8qqaq=dirpe0xb9q8qiLsFr0=vr0=vr0dc8meaabaqaciaacaGaaeqabaqabeGadaaakeaaiiGacqWFdpWCdaqhaaWcbaGaemyAaKMaemOAaOMaem4AaSgabaGaemyraueaaaaa@33CD@ is the standard deviation of the effectiveness. We assume that we can model the mean cost and effectiveness as a linear combination:

μijkC=αC+τkC+ηjC,
 MathType@MTEF@5@5@+=feaafiart1ev1aaatCvAUfKttLearuWrP9MDH5MBPbIqV92AaeXatLxBI9gBaebbnrfifHhDYfgasaacH8akY=wiFfYdH8Gipec8Eeeu0xXdbba9frFj0=OqFfea0dXdd9vqai=hGuQ8kuc9pgc9s8qqaq=dirpe0xb9q8qiLsFr0=vr0=vr0dc8meaabaqaciaacaGaaeqabaqabeGadaaakeaaiiGacqWF8oqBdaqhaaWcbaGaemyAaKMaemOAaOMaem4AaSgabaGaem4qameaaOGaeyypa0Jae8xSde2aaWbaaSqabeaacqWGdbWqaaGccqGHRaWkcqWFepaDdaqhaaWcbaGaem4AaSgabaGaem4qameaaOGaey4kaSIae83TdG2aa0baaSqaaiabdQgaQbqaaiabdoeadbaakiabcYcaSaaa@42FF@

μijkE=αE+τkE+ηjE+(βk+φj)(cijk−μijkC),
 MathType@MTEF@5@5@+=feaafiart1ev1aaatCvAUfKttLearuWrP9MDH5MBPbIqV92AaeXatLxBI9gBaebbnrfifHhDYfgasaacH8akY=wiFfYdH8Gipec8Eeeu0xXdbba9frFj0=OqFfea0dXdd9vqai=hGuQ8kuc9pgc9s8qqaq=dirpe0xb9q8qiLsFr0=vr0=vr0dc8meaabaqaciaacaGaaeqabaqabeGadaaakeaaiiGacqWF8oqBdaqhaaWcbaGaemyAaKMaemOAaOMaem4AaSgabaGaemyraueaaOGaeyypa0Jae8xSde2aaWbaaSqabeaacqWGfbqraaGccqGHRaWkcqWFepaDdaqhaaWcbaGaem4AaSgabaGaemyraueaaOGaey4kaSIae83TdG2aa0baaSqaaiabdQgaQbqaaiabdweafbaakiabgUcaRiabcIcaOiab=j7aInaaBaaaleaacqWGRbWAaeqaaOGaey4kaSIae8NXdy2aaSbaaSqaaiabdQgaQbqabaGccqGGPaqkcqGGOaakcqWGJbWydaWgaaWcbaGaemyAaKMaemOAaOMaem4AaSgabeaakiabgkHiTiab=X7aTnaaDaaaleaacqWGPbqAcqWGQbGAcqWGRbWAaeaacqWGdbWqaaGccqGGPaqkcqGGSaalaaa@5C46@

In equation (2), *α*^C ^is the average cost for the control treatment and τkC
 MathType@MTEF@5@5@+=feaafiart1ev1aaatCvAUfKttLearuWrP9MDH5MBPbIqV92AaeXatLxBI9gBaebbnrfifHhDYfgasaacH8akY=wiFfYdH8Gipec8Eeeu0xXdbba9frFj0=OqFfea0dXdd9vqai=hGuQ8kuc9pgc9s8qqaq=dirpe0xb9q8qiLsFr0=vr0=vr0dc8meaabaqaciaacaGaaeqabaqabeGadaaakeaaiiGacqWFepaDdaqhaaWcbaGaem4AaSgabaGaem4qameaaaaa@3113@ is the additional cost for treatment *k *(by default τ1C
 MathType@MTEF@5@5@+=feaafiart1ev1aaatCvAUfKttLearuWrP9MDH5MBPbIqV92AaeXatLxBI9gBaebbnrfifHhDYfgasaacH8akY=wiFfYdH8Gipec8Eeeu0xXdbba9frFj0=OqFfea0dXdd9vqai=hGuQ8kuc9pgc9s8qqaq=dirpe0xb9q8qiLsFr0=vr0=vr0dc8meaabaqaciaacaGaaeqabaqabeGadaaakeaaiiGacqWFepaDdaqhaaWcbaGaeGymaedabaGaem4qameaaaaa@30A4@ = 0); ηjC
 MathType@MTEF@5@5@+=feaafiart1ev1aaatCvAUfKttLearuWrP9MDH5MBPbIqV92AaeXatLxBI9gBaebbnrfifHhDYfgasaacH8akY=wiFfYdH8Gipec8Eeeu0xXdbba9frFj0=OqFfea0dXdd9vqai=hGuQ8kuc9pgc9s8qqaq=dirpe0xb9q8qiLsFr0=vr0=vr0dc8meaabaqaciaacaGaaeqabaqabeGadaaakeaaiiGacqWF3oaAdaqhaaWcbaGaemOAaOgabaGaem4qameaaaaa@30F8@ is the deviation from the average cost of centre *j*. It is possible to extend this model to allow for covariates [7]. Similarly, in equation (3) *α*^E ^is the average effect, or outcome, for the control treatment and τkE
 MathType@MTEF@5@5@+=feaafiart1ev1aaatCvAUfKttLearuWrP9MDH5MBPbIqV92AaeXatLxBI9gBaebbnrfifHhDYfgasaacH8akY=wiFfYdH8Gipec8Eeeu0xXdbba9frFj0=OqFfea0dXdd9vqai=hGuQ8kuc9pgc9s8qqaq=dirpe0xb9q8qiLsFr0=vr0=vr0dc8meaabaqaciaacaGaaeqabaqabeGadaaakeaaiiGacqWFepaDdaqhaaWcbaGaem4AaSgabaGaemyraueaaaaa@3117@ is the additional effect of treatment *k *(by default τ1C
 MathType@MTEF@5@5@+=feaafiart1ev1aaatCvAUfKttLearuWrP9MDH5MBPbIqV92AaeXatLxBI9gBaebbnrfifHhDYfgasaacH8akY=wiFfYdH8Gipec8Eeeu0xXdbba9frFj0=OqFfea0dXdd9vqai=hGuQ8kuc9pgc9s8qqaq=dirpe0xb9q8qiLsFr0=vr0=vr0dc8meaabaqaciaacaGaaeqabaqabeGadaaakeaaiiGacqWFepaDdaqhaaWcbaGaeGymaedabaGaem4qameaaaaa@30A4@ = 0); ηjC
 MathType@MTEF@5@5@+=feaafiart1ev1aaatCvAUfKttLearuWrP9MDH5MBPbIqV92AaeXatLxBI9gBaebbnrfifHhDYfgasaacH8akY=wiFfYdH8Gipec8Eeeu0xXdbba9frFj0=OqFfea0dXdd9vqai=hGuQ8kuc9pgc9s8qqaq=dirpe0xb9q8qiLsFr0=vr0=vr0dc8meaabaqaciaacaGaaeqabaqabeGadaaakeaaiiGacqWF3oaAdaqhaaWcbaGaemOAaOgabaGaem4qameaaaaa@30F8@ is the deviation from the average effectiveness of centre *j*. *β*_*k *_is a parameter which allows for the relationship between costs and effects, *φ*_*j *_and allows this to vary between clusters. From this model we can define the ICER as:

ICER=τ2Cτ2E.
 MathType@MTEF@5@5@+=feaafiart1ev1aaatCvAUfKttLearuWrP9MDH5MBPbIqV92AaeXatLxBI9gBaebbnrfifHhDYfgasaacH8akY=wiFfYdH8Gipec8Eeeu0xXdbba9frFj0=OqFfea0dXdd9vqai=hGuQ8kuc9pgc9s8qqaq=dirpe0xb9q8qiLsFr0=vr0=vr0dc8meaabaqaciaacaGaaeqabaqabeGadaaakeaacqWGjbqscqWGdbWqcqWGfbqrcqWGsbGucqGH9aqpdaWcaaqaaGGaciab=r8a0naaDaaaleaacqaIYaGmaeaacqWGdbWqaaaakeaacqWFepaDdaqhaaWcbaGaeGOmaidabaGaemyraueaaaaakiabc6caUaaa@3B10@

We also define the probability that the intervention is cost effective (for a given willingness to pay, say *λ*) as Pr(*eλ *– *c *> 0), where *e *is the effect and *c *is the difference in cost.

In our example we shall compare two models, one assuming a normal distribution for the costs and another assuming a gamma distributions for the costs. Both models will assume a Bernoulli distribution for the effectiveness since this was a binary outcome measure. In both the normal and gamma distribution models we shall assume a linear link function (although traditionally a log link function would be used for the gamma distribution in order to ensure that the mean was estimated to be above zero). For the Bernoulli model we shall assume a logit link function since this is the standard model for effectiveness in clinical trials with binary outcome data. None of these models allow for individual (or cluster) level covariates.

In order to complete the Bayesian specification of the model we must assign prior distributions to all the unknown parameters. The particular priors that we shall use are:

ηjE~N(0,σEη2)ηjC~N(0,σCη2)βk~N(0,σβ2)φj~N(0,σφ2)
 MathType@MTEF@5@5@+=feaafiart1ev1aaatCvAUfKttLearuWrP9MDH5MBPbIqV92AaeXatLxBI9gBaebbnrfifHhDYfgasaacH8akY=wiFfYdH8Gipec8Eeeu0xXdbba9frFj0=OqFfea0dXdd9vqai=hGuQ8kuc9pgc9s8qqaq=dirpe0xb9q8qiLsFr0=vr0=vr0dc8meaabaqaciaacaGaaeqabaqabeGadaaakeaafaqabeGacaaabaacciGae83TdG2aa0baaSqaaiabdQgaQbqaaiabdweafbaakiabc6ha+jabd6eaojabcIcaOiabicdaWiabcYcaSiab=n8aZnaaDaaaleaacqWGfbqrcqWF3oaAaeaacqaIYaGmaaGccqGGPaqkaeaacqWF3oaAdaqhaaWcbaGaemOAaOgabaGaem4qameaaOGaeiOFa4NaemOta4KaeiikaGIaeGimaaJaeiilaWIae83Wdm3aa0baaSqaaiabdoeadjab=D7aObqaaiabikdaYaaakiabcMcaPaqaaiab=j7aInaaBaaaleaacqWGRbWAaeqaaOGaeiOFa4NaemOta4KaeiikaGIaeGimaaJaeiilaWIae83Wdm3aa0baaSqaaiab=j7aIbqaaiabikdaYaaakiabcMcaPaqaaiab=z8aMnaaBaaaleaacqWGQbGAaeqaaOGaeiOFa4NaemOta4KaeiikaGIaeGimaaJaeiilaWIae83Wdm3aa0baaSqaaiab=z8aMbqaaiabikdaYaaakiabcMcaPaaaaaa@68D8@

All other parameters are assumed to follow a normal distribution with large variance.

#### 1.2 Non-parametric bootstrapping

We can apply the bootstrap to the model defined by equation (1) whilst allowing both for the relationship between the cost and the effectiveness and for the non-independence of the cost and the effectiveness due to clustering of the data. This method has the advantage of not assuming a specific distribution for either the cost or the effectiveness [19].

The following algorithm will construct a bootstrap sample of *K *replications with *m *clusters each of size *w*. However this particular algorithm is only applicable in the situation where each cluster is of the same size; alternative algorithms are appropriate when this assumption is not true [19,24]. The relationship between costs and effectiveness is retained due to their joint sampling.

#### Bootstrap algorithm

1. For the observed data set estimate the ICER, say ICER^0^;

2. For *i *in 1 to *K*

a. For *j *in 1 to *m*

i. Randomly select (with replacement) a cluster centre, say *k*_*j*_

ii. Within that cluster randomly select (with replacement) *w *sets of costs and effectiveness, these must be selected together in order to preserve the relationship between costs and effectivess.

b. Estimate the ICER on the basis of the bootstrap sample constructed in part *a *(say ICER^*i*^), as the difference between treatment and control groups in mean costs, divided by the difference in mean outcomes.

Confidence intervals can then be constructed for the ICER in various ways [22]. We shall use the bias corrected accelerated percentile method [17]. We also estimated the probability that the intervention was cost effective. This was the proportion of iterations in which the effect, multiplied by the corresponding willingness to pay per effect, was greater than the cost difference.

### 2. Regression-based models of net benefits

The calculation of net benefits reduces costs and effectiveness to a single variable which can be used in standard regression analyses [5]. We define the net benefit (*nb*) as,

*nb*_*ijk *_= *e*_*ijk*_*λ *– *c*_*ijk*_,

Where, as before, *e*_*ijk *_is the effectiveness on the ith person in the jth cluster in arm *k*, *λ *is the money society would be willing to pay for a unit of effectiveness, and *c*_*ijk *_is the cost. Net benefit is expressed in monetary terms and so is also called net monetary benefit.

In a standard simple linear regression model with net benefit as the outcome variable and trial arm as the explanatory variable, the regression coefficient for the treatment term represents the incremental net benefit attributable to the intervention, for that level of willingness to pay. Willingness to pay is explored for a range of levels because it is usually not known. To estimate the corresponding incremental net benefit, 95% confidence limits and P values, a separate regression analysis is done at each willingness to pay level.

The intervention is defined to be cost effective, at a given willingness to pay level, if the corresponding incremental net benefit is greater than zero. Therefore the probability that the intervention is cost effective at a given willingness to pay level is the probability that the incremental net benefit is greater than zero. If the coefficient is greater than zero, then the probability that the intervention is cost effective is one minus half the one sided P value for the treatment term. If the coefficient is less than zero, then the probability that the intervention is cost effective is half the one sided P value. In Bayesian models the probability that the intervention is cost effective is the predictive probability that the net benefit is greater than zero. If the model is estimated using the Markov chain Monte Carlo method this is simply estimated as the proportion of iterations for which the incremental net benefit is greater than zero.

The ICER and its confidence limits can also be estimated from these regression results because the values of *λ *at which the 95% confidence intervals of the incremental net benefit estimate are equal to zero are thus the 95% confidence intervals of the ICER [5]. These can be estimated from the estimated incremental net benefits, and their confidence limits.

Net benefit regression has previously been used to analyse cost effectiveness data from multi-centred trials [4,9], and can be adapted to account for cluster randomization in various ways. We detail three possible methods.

#### 2.1 Least squares regression of net benefits with robust estimates of standard errors

The standard error from the traditional regression model will be inaccurate and lead to an underestimation of the standard errors of the parameter estimates, but it is possible to compensate for this by using the Huber-White sandwich estimator [17]. The Huber-White sandwich estimator accounts for the non-independence of observations within each cluster. However, because it adjusts the standard errors post estimation the likelihood-ratio test statistics are not applicable.

#### 2.2 Least squares regression of net benefits with a hierarchical model

Hierarchical (multi-level) linear regression models account for clustering of net benefits by modelling individuals at the first level and clusters at the second. We used Stata's xtmixed procedure [17] to specify a hierarchical model with net benefit as outcome, with trial arm as explanatory variable and with coefficients and intercepts varying randomly between clusters. In particular, we consider a model of the form:

*nb*_*ijk *_= *μ*_*ijk *_+ *ε*_*ijk*_

*μ*_*ijk *_= *μ *+ *τ*_*k *_+ *θ*_*j *_+ *υ*_*jk*_

θj~N(0,σθ2)
 MathType@MTEF@5@5@+=feaafiart1ev1aaatCvAUfKttLearuWrP9MDH5MBPbIqV92AaeXatLxBI9gBaebbnrfifHhDYfgasaacH8akY=wiFfYdH8Gipec8Eeeu0xXdbba9frFj0=OqFfea0dXdd9vqai=hGuQ8kuc9pgc9s8qqaq=dirpe0xb9q8qiLsFr0=vr0=vr0dc8meaabaqaciaacaGaaeqabaqabeGadaaakeaaiiGacqWF4oqCdaWgaaWcbaGaemOAaOgabeaakiabc6ha+jabd6eaojabcIcaOiabicdaWiabcYcaSiab=n8aZnaaDaaaleaacqWF4oqCaeaacqaIYaGmaaGccqGGPaqkaaa@3ABD@

vjk~N(0,σjk2)
 MathType@MTEF@5@5@+=feaafiart1ev1aaatCvAUfKttLearuWrP9MDH5MBPbIqV92AaeXatLxBI9gBaebbnrfifHhDYfgasaacH8akY=wiFfYdH8Gipec8Eeeu0xXdbba9frFj0=OqFfea0dXdd9vqai=hGuQ8kuc9pgc9s8qqaq=dirpe0xb9q8qiLsFr0=vr0=vr0dc8meaabaqaciaacaGaaeqabaqabeGadaaakeaacqWG2bGDdaWgaaWcbaGaemOAaOMaem4AaSgabeaakiabc6ha+jabd6eaojabcIcaOiabicdaWiabcYcaSGGaciab=n8aZnaaDaaaleaacqWGQbGAcqWGRbWAaeaacqaIYaGmaaGccqGGPaqkaaa@3CEB@

Where *μ *is the overall mean net benefit, *τ*_2 _is the additional net benefit associated with the treatment arm (by default *τ*_2 _= 0), *θ*_*j *_is the deviation in net benefit due to the *j*th clusters centre and *v*_*j*,*k *_is the deviation in net benefit in centre *j *(by default all *v*_1,*k *_are zero).

#### 2.3 Net benefit regression with a Bayesian hierarchical model

Spiegelhalter [24] described a Bayesian two level linear regression model for comparing continuous outcomes in cluster randomized trials, defined as follows:

*nb*_*ijk*_~ *N *(*μ *_*ijk*_, *σ*^2^),

*μ *_*ijk *_= *μ *+ *τ *_*k *_+ *θ*_*j*_,

Where, *μ *is the mean net benefit for the control arm; *τ*_2 _is the additional net benefit associated with the treatment arm (by default *τ*_1 _= 0); and *θ*_*j *_is the deviation in net benefit due to the *j*th clusters centre. In order to complete the model description we shall use the following prior distributions:

θj~N(0,σθ2)
 MathType@MTEF@5@5@+=feaafiart1ev1aaatCvAUfKttLearuWrP9MDH5MBPbIqV92AaeXatLxBI9gBaebbnrfifHhDYfgasaacH8akY=wiFfYdH8Gipec8Eeeu0xXdbba9frFj0=OqFfea0dXdd9vqai=hGuQ8kuc9pgc9s8qqaq=dirpe0xb9q8qiLsFr0=vr0=vr0dc8meaabaqaciaacaGaaeqabaqabeGadaaakeaaiiGacqWF4oqCdaWgaaWcbaGaemOAaOgabeaakiabc6ha+jabd6eaojabcIcaOiabicdaWiabcYcaSiab=n8aZnaaDaaaleaacqWF4oqCaeaacqaIYaGmaaGccqGGPaqkaaa@3ABD@

*τ*_2 _~ *N*(0,100000)

*μ *~ *N*(0,100000)

σθ2~U(0,100000)
 MathType@MTEF@5@5@+=feaafiart1ev1aaatCvAUfKttLearuWrP9MDH5MBPbIqV92AaeXatLxBI9gBaebbnrfifHhDYfgasaacH8akY=wiFfYdH8Gipec8Eeeu0xXdbba9frFj0=OqFfea0dXdd9vqai=hGuQ8kuc9pgc9s8qqaq=dirpe0xb9q8qiLsFr0=vr0=vr0dc8meaabaqaciaacaGaaeqabaqabeGadaaakeaaiiGacqWFdpWCdaqhaaWcbaGae8hUdehabaGaeGOmaidaaOGaeiOFa4NaemyvauLaeiikaGIaeGimaaJaeiilaWIaeGymaeJaeGimaaJaeGimaaJaeGimaaJaeGimaaJaeGimaaJaeiykaKcaaa@3D1D@

This model must be estimated separately for each level of willingness to pay. It could be extended by including an additional random effect of incremental net benefit by centre.

## Results

### Joint models of cost and effect

**Figure 2:**
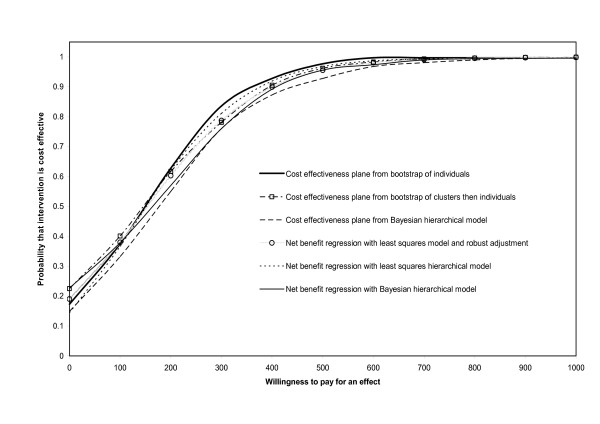
Cost effectiveness acceptability curve: probability that the intervention was cost effective for different levels of willingness to pay and with different analytic methods.

The joint models were fitted using WinBUGS. In particular we ran the Bernoulli-Normal and Bernoulli-Gamma models with random intercept defined by equations 2 and 3. We ran the models for 10,000 iterations, using the first 5,000 as burn-in and the remaining 5,000 were thinned at an interval of 5 in order to give an approximately independent sample. We found that the best fitting model, as indicated by the smallest deviance information criterion, was the Bernoulli-Gamma model including *φ*_*j*_. This model gave an ICER of 189 (95% CI -194 to 647) (Table 1) with probabilities of being cost effective as shown in Figure 2. All of these models gave similar probabilities of being cost effective.

**Table 1:** Incremental cost effectiveness ratios (ZAR). Incremental cost effectiveness ratios (in South African Rand)

**Analytic method**	**ICER**	**95% confidence limits**
**Cost effectiveness plane**		
Bootstrapping individuals	150	-143, 489
Bootstrapping clusters then individuals	150	-918, 217
Bayesian hierarchical model	189	-194, 647
		
**Linear regression of net benefits**		
Least squares model without adjustment	154	-162, 481
Least squares model with robust adjustment	154	-257, 575
Least squares hierarchical model	155	-244, 568
Bayesian hierarchical model	157	-282, 600

ICER incremental cost effectiveness ratio.

The non-parametric bootstrap was carried out with 1,000 bootstrap replications and gave an ICER of 150 (95% CI -918 to 217), the results are shown in Table 1 and in Figure 2. Applying the bootstrap while ignoring the clusters and simply randomly selecting individuals and costs resulted in the same estimated ICER but with much narrower confidence intervals. These results are summarised in Table 1 and Figure 2. The long left tail of the ICER distribution was due to bootstrap samples that produced moderate (negative) differences in costs accompanying small effects, resulting in large negative ICERs.

### Net benefit models

**Figure 3:**
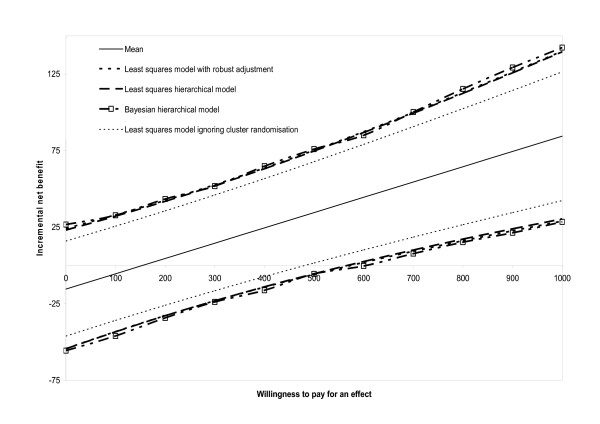
Incremental net benefit at different levels of willingness to pay per unit of effect: 95% confidence limits estimated with different regression models.

The standard linear regression model gave an estimated ICER of 154 (95% CI -162 to 481); once the robust standard errors were taken into account the confidence interval for the ICER increased to (-257 to 575). The least squares hierarchical model resulted in an estimated ICER of 155 (95% CI -244 to 568) and the Bayesian hierarchical model had an estimated ICER of 157 (95% CI -282,600). These results are contained in Table 1 and Figures 2 and 3.

## Discussion

We have demonstrated how individual cost and outcome data from cluster randomized trials can be analysed in various ways with widely available software. In our example different appropriate methods produced similar results. Predictably, adjusting for clustering resulted in greater uncertainty about ICERs, and lower probabilities that the intervention was cost effective, compared to methods that ignored clustering. Of all our results, the ICER confidence intervals from 2 stage bootstrapping differed most from confidence intervals from other methods. ICER point estimates were similar (150–157) except for the Bayesian hierarchical joint model of costs and effects (189) because the latter assumed a gamma distribution of costs.

To be able to interpret ICER confidence intervals that include zero, one needs to plot uncertainties about differences in costs and effects on a cost effectiveness plane (Figure 1). This is because, if the cost effectiveness ellipse extends to non-adjacent quadrants, a negative ICER confidence interval is uninterpretable. This is because it combines information about dominant situations with greater costs and worse outcomes, and situations with lower costs and better outcomes [5]. But in this example the negative ICER represented the latter situation only (Figure 1). That is, even if society was willing to pay up to these amounts (the lower confidence limits in Table 1) to *avoid *one unit of effect, the intervention would still be cost effective because of cost saving. So the lower confidence limit, which is reassuring, is here of less interest than the upper confidence limit, which shows how much might have to be paid for an effect.

Our example of one trial has limited generalizability, which would be enhanced by comparing these methods using different data from other trials and from simulations. Problems could potentially occur with fewer clusters or with varying numbers of individuals per cluster. For example, Flynn and Peters used simulated data to show that, with 24 or fewer clusters per arm, Stata's bootstrap ICER estimates may be spuriously precise [3]. They also found that Stata's robust adjustment performed better than its bootstrap procedures in estimating cost differences [23]. Net benefit regression may be invalid with small samples if net benefits are not normally distributed. In this example, however, estimates from net benefit regression were similar to nonparametric bootstrap estimates, as predicted by the central limit theorem. The bootstrap methods we describe may be inappropriate if cluster sizes vary [3,20], in which case more sophisticated methods might be needed [24]. Net benefit regression models and Nixon's and Thompson's two dimensional model [7] need not assume equal cluster size. Nixon's and Thompson's model has several other advantages. It can accommodate various cost distributions, does not need to be repeated at different willingness to pay levels, does not need separate regression analyses to estimate ICER confidence intervals, and can produce the cost effectiveness ellipse needed to interpret a negative ICER. Two stage bootstrapping has similar advantages, but we found its results to be unreliable over repeated analyses, even with 10000 iterations. A pragmatic approach is to check the robustness of the primary analysis by also using another method, especially if there are few clusters or if their sizes vary.

## Competing interests

The author(s) declare that they have no competing interests.

## Authors' contributions

MOB conceived of the paper, chose the statistical methods, carried out the Stata analyses, and was lead writer of the paper and co-investigator of the randomised trial and economic evaluation. LF carried out Stata analyses and was principal investigator of the trial and the economic evaluation. AC helped choose the Bayesian models and carried out and described the WinBUGS analyses. MM was a co-investigator on the economic evaluation. All authors contributed to writing the paper and read and approved the final manuscript.
